# A CRISPR/Cas9-mediated screen identifies determinants of early plasma cell differentiation

**DOI:** 10.3389/fimmu.2022.1083119

**Published:** 2023-01-05

**Authors:** Ermeng Xiong, Oliver Popp, Claudia Salomon, Philipp Mertins, Christine Kocks, Klaus Rajewsky, Van Trung Chu

**Affiliations:** ^1^ Immune Regulation and Cancer, Max-Delbrück-Center for Molecular Medicine in the Helmholtz Association (MDC), Berlin, Germany; ^2^ Proteomics platform, Max-Delbrück-Center for Molecular Medicine in the Helmholtz Association (MDC) and Berlin Institute of Health (BIH), Berlin, Germany; ^3^ Genome Engineering & Disease Models, Max-Delbrück-Center for Molecular Medicine in the Helmholtz Association (MDC), Berlin, Germany

**Keywords:** CRISPR/Cas9, screen, gene knockout, regulators, determinants of plasma cell differentiation, antibody-secreting cells, plasma cell differentiation, plasmablasts

## Abstract

**Introduction:**

The differentiation of B cells into antibody-secreting plasma cells depends on cell division-coupled, epigenetic and other cellular processes that are incompletely understood.

**Methods:**

We have developed a CRISPR/Cas9-based screen that models an early stage of T cell-dependent plasma cell differentiation and measures B cell survival or proliferation versus the formation of CD138+ plasmablasts. Here, we refined and extended this screen to more than 500 candidate genes that are highly expressed in plasma cells.

**Results:**

Among known genes whose deletion preferentially or mostly affected plasmablast formation were the transcription factors Prdm1 (BLIMP1), Irf4 and Pou2af1 (OBF-1), and the Ern1 gene encoding IRE1a, while deletion of XBP1, the transcriptional master regulator that specifies the expansion of the secretory program in plasma cells, had no effect. Defective plasmablast formation caused by Ern1 deletion could not be rescued by the active, spliced form of XBP1 whose processing is dependent on and downstream of IRE1a, suggesting that in early plasma cell differentiation IRE1a acts independently of XBP1. Moreover, we newly identified several genes involved in NF-kB signaling (Nfkbia), vesicle trafficking (Arf4, Preb) and epigenetic regulators that form part of the NuRD complex (Hdac1, Mta2, Mbd2) to be required for plasmablast formation. Deletion of ARF4, a small GTPase required for COPI vesicle formation, impaired plasmablast formation and blocked antibody secretion. After Hdac1 deletion plasmablast differentiation was consistently reduced by about 50%, while deletion of the closely related Hdac2 gene had no effect. Hdac1 knock-out led to strongly perturbed protein expression of antagonistic transcription factors that govern plasma cell versus B cell identity (by decreasing IRF4 and BLIMP1 and increasing BACH2 and PAX5).

**Discussion:**

Taken together, our results highlight specific and non-redundant roles for Ern1, Arf4 and Hdac1 in the early steps of plasma cell differentiation.

## Introduction

Upon challenging with T cell-independent antigens through Toll-like receptors or the B cell receptor, B cells proliferate and directly differentiate into short-lived plasma cells secreting IgM antibodies, generally unmutated and of low affinity, while long-lived plasma cells producing antibodies with high affinity may be mostly generated through T cell-dependent responses in germinal centers (GCs) (1, 2). In the GC response, GC B cells undergo massive proliferation, heavy chain class-switch recombination, and somatic hypermutation. T follicular helper (T_FH_) cells in GCs express CD40 ligand (CD40L) on the cell surface and secrete cytokines such as IL-21, which, through CD40 and IL-21 receptors on the surface of GC B cells, activate multiple signaling pathways to induce the terminal differentiation of GC B cells into plasma cells (3–6).

Plasma cell differentiation requires profound transcriptional and epigenetic reprogramming and is linked to cell division (7). On the one hand, transcriptional reprogramming is accomplished through the interplay of opposing transcriptional networks: PAX5, BCL6, BACH2 and low levels of IRF4 govern B cell and GC B cell fates whereas high levels of IRF4, BLIMP1 and XBP1 dictate the plasma cell program. Through repression of each other’s expression, the latter gain functional dominance over the former during plasma cell differentiation (8, 9). On the other hand, epigenetic reprogramming comprises DNA methylation/and demethylation, histone modification and chromatin remodeling, which ultimately determine gene expression and thereby have important roles in plasma cell differentiation (7). However, the mechanisms by which epigenetic factors regulate plasma cell differentiation are largely unknown. The polycomb repressive complex 2 has been implicated in repression of B cell genes during plasma cell differentiation (10), and it has been shown that BLIMP1 recruits EZH2, a catalytic subunit of polycomb repressive complex 2, to distinct DNA regions to repress genes that govern B cell identity (11–13).

A morphological hallmark of plasma cells is a highly developed secretory apparatus comprising an expanded and specialized network of endoplasmic reticulum (ER) where massive quantities of antibodies are co-translationally produced, folded and modified (14). Accumulation of unfolded or misfolded proteins in the ER lumen triggers the unfolded protein response (UPR), which is regulated by three ER trans-membrane sensors in metazoan cells: serine/threonine-protein kinase/endoribonuclease inositol-requiring enzyme 1α (IRE1α), activating transcription factor 6 (ATF6) and PKR-like ER kinase (PERK) (15). In plasma cells, the ATF6 and PERK pathways are functionally suppressed, while the IRE1α pathway remains active, endowing plasma cells with a specialized UPR which is not well understood (16, 17).

Here, we extended and refined a previously established CRISPR/Cas9-based screen (18, 19), combining CRISPR/Cas9-mediated gene knock-out with an *in vitro* B cell culture system that models T cell-dependent plasma cell formation through co-culture of B cells with mouse fibroblasts that stably express mouse CD40L and BAFF (40LB cells) (20). Our screening system allows the discrimination between effects on B cell survival or proliferation on the one hand, and early plasmablast (PB) formation on the other. While the majority of our hits affected B cell survival or proliferation excluding their further analysis (133 of 568 genes), we identified 24 genes that preferentially or mostly affected PB formation, half of which were previously unknown.

## Materials and methods

### Mice

Rosa26-Cas9iGFP mice were generated previously (21). C57BL/6N mice were obtained by local breeding. Blimp1-YFP transgenic mice were imported from the Jackson Laboratory (JAX stock #008828; B6.Cg-Tg(Prdm1-EYFP)1Mnz/J) (22, 23). Animal experiments were approved by the Institution Animal Care and Use Committee.

### Cell culture

B cells were isolated from R26-Cas9iGFP and C57BL/6N mice as previously described (18). Cas9 (GFP^+^)-expressing B cells were mixed with wildtype (WT, GFP^-^) B cells at a ratio of 50:50 and cultured in DMEM (Gibco) supplied with 10% FCS (Gibco), 2 mM L-Glutamine, 2 mM Sodium Pyruvate, 2mM HEPES (Gibco), 1 x NAA (Gibco), beta-mercaptoethanol (Sigma), and 10μg/ml Gentamicin (Lonza), 5 μg/ml anti-CD40 antibody (BD Biosciences, Cat #553721, RRID:

AB_395006) and 20 ng/ml of IL-4 (PerproTech) for 48 h before retroviral transduction at a density of 10^6^ cells/ml. Retroviral Plat-E packaging cells were maintained in DMEM (Gibco) supplied with 10% FCS (Gibco), 2 mM L-Glutamine (Gibco), and 2 mM Sodium Pyruvate (Gibco). 40LB feeder cells stably expressing mouse BAFF and CD40L (20), were maintained in complete DMEM medium. To prepare the feeder layer, 40LB feeder cells were irradiated with 18 Gγ and plated at 5x10^4^ cells/cm^2^.

### sgRNA design and 96-well cloning approach

Approximately 570 genes highly expressed in plasma cells, were selected. These genes correspond to biological categories such as DNA binding, chromatin remodeling, ER/UPR, Ras superfamily, signaling and secretory pathways (**Supplementary Table 1**). 1-4 sgRNAs per gene were designed using CrispRGold program (**Supplementary Table 2**) (18, 19). Forward and reverse oligos of each sgRNA were mixed and phosphorylated individually. Annealed duplexes were then cloned into the MSCV_hU6_CcdB_PGK_Puro_T2A_BFP vector and plasmids were produced in 96-well plates as previously described (18).

### High-throughput retroviral production and transduction

The protocol for high-throughput retroviral production was previously described (18). Briefly, Plat-E cells were seeded at 2.2 x 10^4^ cells/100 μl medium in each well of 96-well plates at day 0. One day later, the cells were transfected with 100 ng DNA and Fugene®HD transfection reagents (Promega). Retrovirus-containing supernatant was collected 48 h and 72 h after transfection into 96 deep-well plates. 48 h after activation, GFP^+^/GFP^-^ mixed B cells were spin-transduced with retroviral particles expressing sgRNAs in a 96-well plate in the presence of 16 μg/ml Polybrene (Sigma), 5 μg/ml anti-CD40 antibody (BD Biosciences, Cat# 553721, RRID : AB_395006) and 20 ng/ml IL-21 (PeproTech). The 96-well plates were placed in a 37^°^C incubator and on the next day the transduced GFP^+^/GFP^-^ B cells were transferred into 96-well 40LB feeder cell plates in the presence of 20 ng/ml IL-21 and were selected with 2.5 μg/ml puromycin (Sigma). The transduced GFP^+^/GFP^-^ B cells were harvested and analyzed at different time points by using a BD Fortessa with a 96-well HTS unit.

### Survival/proliferation and differentiation scores

Pre-gating on the BFP^+^ population (stably expressing sgRNAs), the frequency of GFP^+^ (Cas9-expressing) and GFP^-^ (WT) B cells was measured. Survival/proliferation: The fold change was calculated as the frequency of GFP^+^ B cells on day 6 normalized to the frequency of GFP^+^ B cells at day 2. Differentiation: The fold change was calculated as the frequency of CD138^+^GFP^+^ (Cas9-expressing) PBs divided by the frequency of CD138^+^GFP^-^ (WT) PBs.

### Generation of MSCV retroviral vectors for cDNA over-expression

Coding (cDNA) sequences of XBP1s, IRE1α^WT^, IRE1α^K599A^, IRE1α^K907A^ and IRE1α^dmutant^ were ordered from IDT and cloned into AgeI/EcoRI sites of the MSCV-IRES-mCherry vector (Addgene, #52114). For co-transduction, the activated B cells were spin-transduced with both retroviral particles expressing sgRNA (BFP^+^) targeting either *Rosa26* or *Ern1* gene, and cDNA (mCherry^+^) at a ratio of 1:1. To detect XBP1s mRNA, BFP^+^mCherry^+^ B cells were sorted four days post transduction. To quantify CD138^+^ PBs, the transduced cells were analyzed six days post co-transduction.

### Antibodies, FACS analysis and sorting

For FACS analysis, the transduced B cells were collected and blocked with TruStain FcX™ antibody (Biolegend, Cat #101320, RRID: AB_1574975) for 10 min. The single cell suspension was stained with Brilliant Violet 785™ anti-mouse/human CD45R/B220 (BioLegend, Cat #103246, RRID: AB_2563256), Brilliant Violet 605™ anti-mouse CD19 (Biolegend, Cat #115540, RRID: AB_2563067) and APC anti-mouse CD138 (Biolegend, Cat #142506, RRID: AB_10962911) antibodies for 15 min. The stained cells were washed with FACS buffer (PBS/1%BSA) and analyzed by BD Fortessa. PI (Propidium Iodide, Biolegend, Cat #421301) and DAPI (4’,6-Diamidino-2-Phenylindole, Dilactate, Biolegend, Cat #422801) were used to exclude dead cells. FACS data was analyzed by FlowJo software (v10.6, BD Life Sciences). For cell sorting, the stained B cells were sorted into 15 ml Falcon tubes and centrifuged prior to DNA, RNA and protein isolation.

Intracellular staining for IgG1, the transduced Cas9-expressing B cells were stained with surface antibodies and LIVE/DEAD™ Fixable Near-IR Dead Cell Stain Kit (Invitrogen, Cat #L34976) to exclude dead cells. The stained cells were fixed and permeabilized with Cytofix/Cytoperm™ solution (BD Biosciences, Cat #554722) for 20 min at RT and stained with PE anti-mouse IgG1 (BD Biosciences, Cat #550083, RRID: AB_393553) in 1x Perm/Wash Buffer (BD Biosciences, Cat #554723) for 30 min at RT. Finally, these cells were washed and analyzed by BD Fortessa.

### Semiquantitative-PCR for XBP1s detection

To measure expression levels of Xbp1s mRNA, total RNA was isolated from sorted B cells using RNAeasy kits (Qiagen) and reverse-transcribed into cDNA using SuperScript™ III kit (Thermo Fisher Scientific). *Xbp1* transcript was amplified from cDNA by PCR using Platinum SuperFi PCR Master Mix (Thermo Fisher Scientific) with forward (5’-ACACGCTTGGGAATGGACAC) and reverse (5’-CCATGGGAAGATGTTCTGGG) primers and following PCR conditions: 98^0^C for 2 min; 25 cycles (98^0^C for 10s, 60^0^C for 30s, 72^0^C for 30s) and 72^0^C for 5 min. *Gapdh* was used as a loading control. Xbp1u and Xbp1s were separated on 2.5% agarose gels.

### Western blotting

Sorted B cells and CD138^+^ PBs were lysed in RIPA buffer (20mM Tris-HCl (pH 7.5), 150mM NACl, 1% NP-40, 0.1% SDS, 0.1% Sodium deoxycholate and 1mM EDTA) supplied with protease inhibitors. B cell lysates were fractionated by SDS-PAGE and transferred to PVDF membrane (GE Healthcare). PERK, ATF6α, IRE1α, ARF4, HDAC1, HDAC2, BLIMP1, IRF4, BACH2 and PAX5 proteins were detected using primary antibodies: rabbit anti-PERK (Cell Signaling Technology, Cat# 3192S, RRID: AB_2095847), rabbit anti-ATF6α (Cell Signaling Technology, Cat# 65880S, RRID: AB_2799696), rabbit anti- IRE1α (Cell Signaling Technology, Cat# 3294S, RRID: AB_823545), rabbit anti-ARF4 (Proteintech, Cat#11673-1-AP, RRID: AB_2058463), mouse anti-HDAC1 (Santa Cruz Biotechnology, Cat# sc-81598, RRID: AB_2118083), mouse anti-HDAC2 (Santa Cruz Biotechnology, Cat# sc-81599, RRID: AB_2118560), mouse anti-BLIMP1 (Novus, Cat# NB600-235, RRID : AB_10595225), rat anti-IRF4 (BioLegend, Cat# 646402, RRID: AB_2280462), rabbit anti-PAX5 (Cell Signaling Technology, Cat# 8970, RRID: AB_10950222), and rabbit anti-BACH2 (Rockland, Cat# 600-401-H35, RRID: AB_2614304), respectively. Beta-Actin and beta-Tubulin were developed with mouse anti-β-Actin (Sigma-Aldrich Cat# A2228, RRID : AB_476697) and rabbit anti-β-Tubulin (Abcam Cat# ab6046, RRID : AB_2210370) as loading controls, respectively. The primary antibodies were developed with HRP conjugated goat anti-rabbit, rabbit anti-mouse or donkey anti-rat IgG antibodies (all from Southern Biotech).

### ELISPOT assay

Enzyme-linked immunospot (ELISPOT) assays were performed to determine the IgG1 antibody secretion capacity of sorted CD138^+^ PBs. Briefly, ~200-1000 CD138^+^ PBs were directly sorted into individual wells of ELISPOT plates (Millipore) coated with goat anti-mouse Ig (Southern Biotech) and incubated at 37^°^C for 2 hours. After washing, the plates were incubated with biotin-conjugated goat anti-mouse IgG1 for 1 h at RT. The plates were developed with AP-conjugated Streptavidin (Roche) for 30 min at RT. Spots were visualized with the AP substrate BCIP/NBT (GeneTex) and images were captured with a CTL-immunospot analyzer (Immunospot). The antibody secreting areas were quantified using ImageJ software v.1.46r (NIH).

### Sample preparation for mass spec LC-MS parameters

TMT proteomics was carried out as previously described (24). Briefly, an amount of 100 µg of each tryptic peptide sample was labelled using randomly assigned channels from TMT10 reagents (Thermo Fisher Scientific). Peptides were fractionated into 24 fractions using an UltiMate 3000 Systems (Thermo Fisher Scientific) and measured on a Q Exactive HF-X and a Q Exactive Plus orbitrap mass spectrometer (Thermo Fisher Scientific) connected to an EASY-nLC system 1200 system (Thermo Fisher Scientific). For analysis, MaxQuant version 1.6.0.13 (25) was used employing MS2-based reporter ion quantitation. Carbamidomethylation was set as a fixed modification and deamidation on asparagine and oxidised methionine as variable modifications. A PIF filter was applied with a threshold of 0.5. For database search, a Uniprot mouse database (2018) plus common contaminants were used.

### Proteome and GO enrichment analysis

For data analysis, corrected log2-transformed reporter ion intensities were used to calculate ratios of treatment over control. The resulting log fold changes were normalized by median-MAD and subjected to a Bland-Altman reproducibility filtering procedure with an alpha value of 0.1 before applying a one-sample moderated t-test (26). P-values were adjusted using the Benjamini-Hochberg procedure. For GO enrichment analysis, sets of up- and down-regulated genes/proteins were enriched for GO biological processes using Metascape software (https://metascape.org) (27).

### Statistical analysis

Statistical tests were performed with Prism 8.0 (GraphPad) using the non-parametric Mann-Whitney test or two-way ANOVA. P values are shown as *P < 0.05, **P < 0.01, ***P < 0.001, ****P < 0.0001. For each experiment, at least three independent experiments were performed. The number of biological replicates for each type of experiment is outlined in the figure legends or shown as data points in figures.

## Results

### CRISPR/Cas9-mediated screen for B cell survival/proliferation and plasma cell differentiation

**Figure 1:**
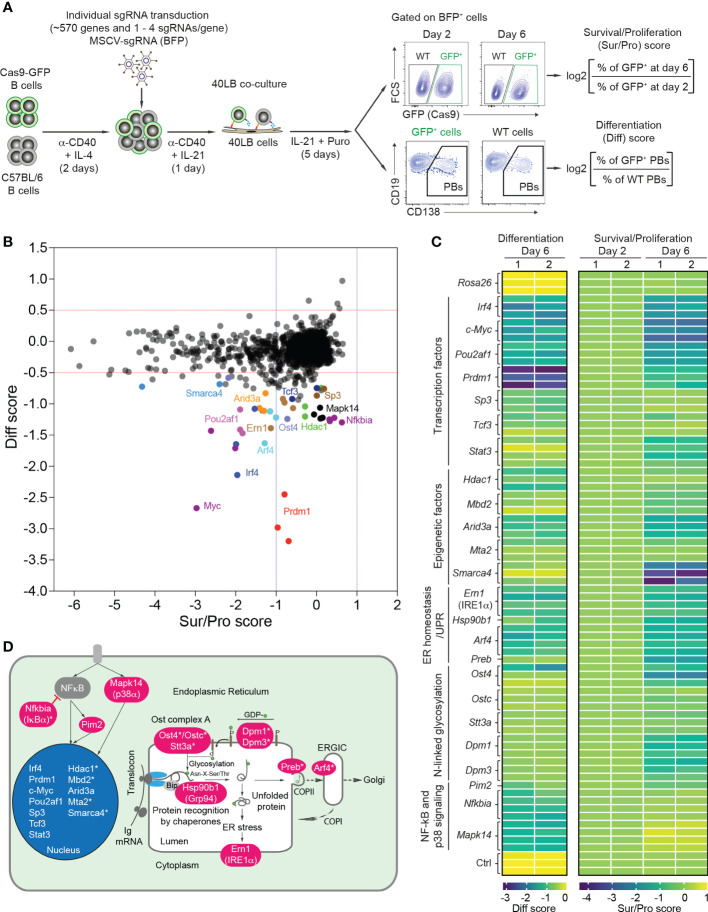
CRISPR/Cas9-mediated screen to differentiate B cell survival/proliferation and plasma cell differentiation. **(A)** Scheme of CRISPR/Cas9-mediated screen in 40LB culture system. Cas9-expressing B cells were isolated from R26-Cas9iGFP/+ mice, mixed with C57BL/6 (WT) B cells at a ratio of 50:50, and activated with α-CD40 antibodies and IL-4 for 2 days. The activated B cells were transduced with retroviral particles expressing specific sgRNAs in 96 well plates. 1 day after transduction, the transduced cells were transferred into 96 well plates containing irradiated 40LB feeder cells and cultured in the presence of IL-21 and puromycin. These cells were analyzed at day 2 and 6 after transduction by flow cytometry. Pre-gating on BFP^+^ (sgRNA) population, survival/proliferation (Sur/Pro) and differentiation (Diff) scores were defined as log2 (fold change). **(B)** Representative correlation graph showing survival/proliferation vs differentiation scores from one of two experiments (n=2 biological replicates). The cutoffs of log2 fold change for differentiation (<-0.5) and survival/proliferation (<-1) scores are indicated with red and blue dashed lines, respectively. **(C)** Heatmaps showing differentiation (left) and survival/proliferation (right) scores for 24 genes affecting PB differentiation. **(D)** 24 genes affecting PB differentiation were mapped to cellular components. Asterisks indicate newly identified genes.

We extended a previously established CRISPR/Cas9-mediated screen (18) to 568 genes that are highly expressed in plasma cells, and involved in transcriptional regulation, chromatin remodeling, ER homeostasis and the UPR, vesicle trafficking, signaling and secretory pathways (**Supplementary Table 1**). Using CrispRGold 1.1, an improved algorithm for sgRNA design (19), we designed 1-4 sgRNAs per gene and cloned them into an MSCV retroviral vector carrying a BFP reporter gene (**Supplementary Table 2**). Retroviral transduction experiments were performed in a 96-well system (18) in which Cas9-expressing B cells were mixed with wild-type (WT) B cells and activated with anti-CD40 antibody and IL-4 for 2 days, followed by IL-21 stimulation for one day and subsequent co-culture in the presence of IL-21 and puromycin on irradiated 40LB feeder cells. These conditions are intended to model T cell-dependent B cell activation in GCs (20). We adapted the read-out to higher throughput by analyzing the frequencies of GFP^+^ B cells and CD138^+^ PBs on day 2 and day 6, and by introducing scores for survival/proliferation (sur/pro) and PB differentiation (diff) (**Figure 1A**). Largely in agreement with our previous small-scale screen (limited to 80 candidate genes) (18), we identified 424 genes to have no effect, 133 genes to have a strong impact on B cell survival/proliferation (sur/pro score <-1) and 24 genes to preferentially or mostly affect CD138^+^ PB differentiation (diff score <-0.5) (**Figures 1B, C**, **Supplementary Figure 1**, **Supplementary Figure 2** and **Supplementary Table 3**). The set of 133 genes important for B cell survival/proliferation reflected the biological processes from which the 568 candidate genes were chosen (**Supplementary Table 1**). The strong effect on B cell survival or proliferation after deletion of many of these genes complicated the analysis of their role in PB formation.

We therefore focused our attention on 24 genes which predominantly or selectively affected PB differentiation (**Figure 1C**). This gene set included the major factors known to be important for plasma cell differentiation: transcription factors (*Prdm1* (BLIMP1), *Irf4*, *Pou2af1* (OBF-1), *c-Myc*, *Sp3*, *Tcf3* (E2A) and *Stat3*) (9, 28–33), epigenetic factor *Arid3a* (34), kinase *Pim2* (35), chaperone *Hsp90b1* (GRP94) (36), p38 kinase *Mapk14* (37), and *Ern1*, encoding IRE1α, the principal UPR ER stress sensor active in plasma cells (38). Further hits, previously unknown to play a role in plasma cell differentiation (indicated with an asterisk in **Figure 1D**), corresponded to the epigenetic factors *Hdac1*, *Mbd2*, *Mta2* (components of Mi-2/nucleosomal remodeling and deacetylase (NuRD) complexes) and *Smarca4*, the glycosylation genes *Ost4*, *Ostc*, *Stt3a*, *Dpm1* and *Dpm3*, the vesicle trafficking genes *Arf4* and *Preb*, and the NF-kB signaling gene *Nfkbia* (**Figures 1C, D** and **Supplementary Table 3**).

### Loss of IRE1α arrests PB formation, independently of XBP1

Plasma cells secrete extraordinarily high amounts of antibodies that must be correctly folded and post-translationally modified. In order to maintain ER homeostasis under these conditions and to adapt to the ensuing ER stress, plasma cells are dependent on the UPR and the ER-associated protein degradation (ERAD) pathway (16, 17). Typically, three distinct but partially overlapping UPR sensor pathways cooperate to maintain protein homeostasis, or induce apoptosis in case of failure to so. However, during LPS-induced terminal B cell differentiation, the UPR is triggered transiently by developmental signals in an anticipatory way (39, 40), while at the plasma cell stage two of the UPR sensor pathways (PERK and ATF6) become functionally suppressed (39, 41), equipping plasma cells with a specialized UPR that relies mostly on IRE1α (16).

**Figure 2:**
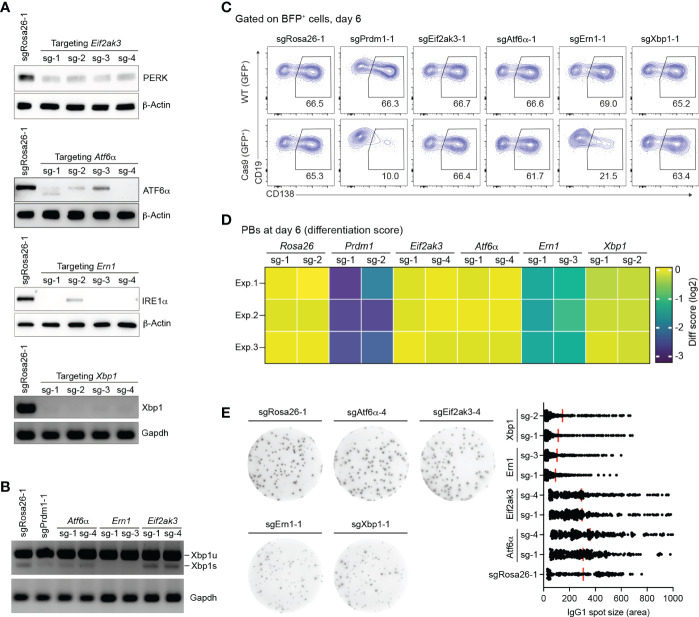
Loss of IRE1α, but not XBP1, arrests PB differentiation. **(A)** Western blots or semiquantitative-PCR assays showing gene KO efficiencies of sgRNAs targeting *Eif2ak3*, *Atf6*α, *Ern1* and *Xbp1* genes. SgRosa26-1 was used as a negative control. Beta-Actin or Grapdh was used as a loading control. **(B)** Xbp1u and Xbp1s mRNAs were measured by semiquantitative-PCR in cells treated with sgRNAs as indicated. **(C)** FACS analysis of PB differentiation, as measured by the expression of CD138. Gates are set on CD138^+^ PBs in WT (GFP^-^, top) and Cas9 (GFP^+^, bottom) cells. **(D)** Heatmap of differentiation score in cells treated with the indicated sgRNAs (n=3 independent experiments). **(E)** Representative ELISPOT images showing antibody secretion in cells treated with the indicated sgRNAs. Graph showing size (area) of IgG1 spots, quantified from a pool of 3 independent experiments.

We validated the effect of gene knockout (KO) for sgRNAs targeting the three known UPR sensors, *Eif2ak3* encoding PERK, *Atf6*α, *Ern1* encoding IRE1α, and additionally *Xbp1* in our *in vitro* screening system. Based on western blotting and semiquantitative-PCR assays, all sgRNAs targeting *Eif2ak3*, *Atf6*α, *Ern1* and *Xbp1* led to high gene KO efficiencies (~80%) in Cas9-expressing B cells 4 days post retroviral transduction (**Figure 2A**). Consistent with previous publications (42, 43) the disruption of IRE1α by CRISPR/Cas9 resulted in a significant reduction of the mRNA encoding the active, spliced form of the transcription factor XBP1, the key regulator of late events in plasma cell differentiation such as the expansion of the secretory apparatus and other organelles that are required in professional secretory cells (44, 45) (**Figure 2B**). *Xbp1* mRNA gets cleaved by the endoribonuclease domain of IRE1α in order to be converted from an inactive, unspliced (XBP1u) to a highly active, spliced form (XBP1s) in an unconventional, cytoplasmic splicing event (46). As expected, inactivation of the UPR sensors ATF6α and PERK using CRISPR/Cas9 was dispensable for B cell survival/proliferation, plasma cell differentiation or antibody secretion (**Figures 2C–E** and **Supplementary Figure 3**). While the percentage of CD138^+^ PBs was dramatically reduced in IRE1α KO B cells, to a level comparable to *Prdm1* KO cells, *Xbp1* KO B cells were unaffected six days post transduction (**Figures 2C, D**). This result is line with XBP1’s known functions in regulating late plasma cell differentiation (expansion of the secretory apparatus for massive antibody secretion) (9, 44, 45). Notably, we observed ~50% reduction in the absolute number of *Prdm1* and IRE1α KO B cells at day 6 after transduction, suggesting that a blockage in PB differentiation inhibits the survival or proliferation of the activated B cells (**Supplementary Figure 3**). By contrast, consistent with the well-known role of the IRE1α-XBP1 axis for antibody secretion at a later stage in plasma cell differentiation (16), both IRE1α and *Xbp1* KO B cells showed impaired secretion of IgG1 antibodies in an ELISPOT assay (**Figure 2E**). Taken together, our results suggest that IRE1α plays a previously unknown role in early PB differentiation that is independent of XBP1.

In order to find out whether XBP1s and its downstream effectors are active in our *in vitro* cell culture system, we performed quantitative proteomics for activated B cells 4 days post transduction in control (sgRosa26-1) and IRE1α KO cells (sgErn1) (**Supplementary Figure 4A**). Among the 95 significantly down-regulated proteins were XBP1 and many known XBP1s target proteins (such as BHLHA15 (MIST-1), EDEMs, TXNDC11, DNAJC3, CDC6, HSPA13, HYOU1 and CRELD2) (**Supplementary Figure 4B** and **Supplementary Table 4**). Accordingly, the set of 95 down-regulated proteins was strongly enriched for the UPR pathway (**Supplementary Figure 4C**). These data clearly showed that the XBP1s-mediated UPR is turned on in activated B cells in our cell culture system and becomes disturbed in IRE1α KO cells. Taken together with the observation that XBP1 is neither essential for survival or proliferation, nor affecting early PB formation (**Supplementary Table 3**) in our *in vitro* B cell culture, our results suggest that XBP1 is mediating UPR-related functions in activated B cells that are not critical for PB formation.

**Figure 3:**
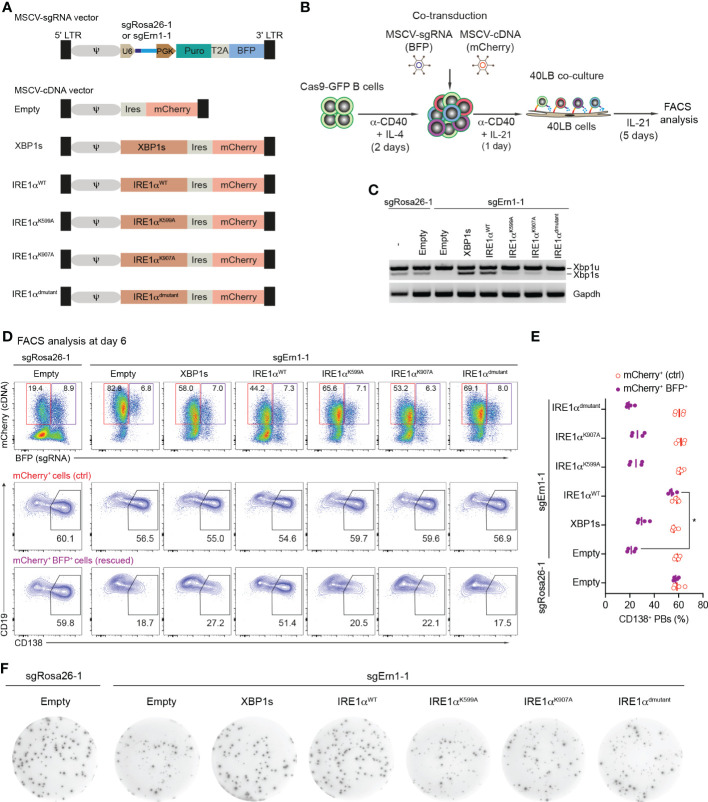
Over-expression of *Ern1*/IRE1α, but not *Xbp1* cDNA rescues PB formation in *Ern1* KO cells. **(A)** Scheme of MSCV-mCherry vectors expressing cDNAs for XBP1s, IRE1α^WT^, IRE1α^K599A^, IRE1α^K907A^, or IRE1α^dmutant^ (double mutant), respectively. As a negative control, empty vector was used. **(B)** Experimental scheme of co-transduction for activated Cas9-expressing B cells transduced with combinations of retroviral particles expressing an sgRNA (BFP^+^), targeting either *Rosa26* (sgRosa26-1) or *Ern1* (sgErn1-1) genes, and a cDNA (mCherry^+^). **(C)** Xbp1u and Xbp1s mRNAs were quantified 4 days post transduction with the indicated sgRNA/cDNA combinations by semiquantitative-PCR of sorted BFP^+^mCherry^+^ cells. **(D)** FACS analysis of the percentage of PB differentiation, indicated by the expression of CD138, at day 6 after co-transduction in mCherry^+^ (ctrl, red) or BFP^+^mCherry^+^ (rescued, magenta) cell populations. **(E)** Quantification of the frequency of CD138^+^ PBs corresponding to the experiment shown in **(D)** plus three biological repeat experiments (statistically significant difference is indicated: Mann-Whitney U-test, *, p=0.02; each dot represents one independent experiment; vertical line indicates median). **(F)** ELISPOT images showing antibody secretion in the Cas9-expressing B cells transduced with the indicated retroviral combinations. Data are representative of three independent experiments (see also **Supplementary Figure 5B**).

We wondered whether overexpression of either XBP1s or catalytically inactive mutants of IRE1α would be able to overcome the disturbed PB formation in IRE1α-deficient B cells. To this end we generated MSCV-mCherry retroviral vectors expressing coding sequences of XBP1s, IRE1α wildtype (IRE1α^WT^), and IRE1α mutants harboring a K599A mutation in the kinase domain (IRE1α^K599A^, “kinase dead”), a K907A mutation in the endoribonuclease domain (IRE1α^K907A^, “endoribonuclease dead”) or both mutations (IRE1α^dmutant^), respectively. As a control, empty vector was used (**Figure 3A** and **Supplementary Figure 5A**). Next, we co-transduced Cas9-expressing B cells with a combination of retroviral particles encoding sgRNAs (BFP^+^ - targeting either the *Rosa26* or *Ern1* genes) and cDNAs coding for XBP1s and IRE1α variants (mCherry^+^) (**Figure 3B**). Four days post co-transduction, BFP^+^mCherry^+^ cells were sorted and the presence of the XBP1s transcription factor was measured using semiquantitative-PCR. As expected, we detected high levels of *Xbp1s* mRNA in the KO cells after expression of XBP1s or IRE1α^WT^ cDNA, whereas all three IRE1α mutants inefficiently rescued XBP1s expression, indicating that both the kinase and endoribonuclease domains of IRE1α are required for the production of XBP1s (**Figure 3C**). In order to address whether the overexpression of the indicated cDNAs can restore PB differentiation in IRE1α KO B cells, we analyzed the frequencies of CD138^+^ PBs among mCherry^+^ (cDNA only, ctrl) and BFP^+^mCherry^+^ (rescued) cell populations. Only overexpression of IRE1α^WT^ fully restored PB formation, while overexpression of IRE1α mutants and XBP1s was insufficient (**Figures 3D, E**). In contrast to this and as expected, antibody secretion in IRE1α KO cells could be fully rescued by overexpressing both XBP1s and IRE1α^WT^ cDNAs, but not IRE1α mutants (**Figure 3F** and **Supplementary Figure 5B**). Taken together, our results suggest that IRE1α acts independently of XBP1 in promoting early PB differentiation, whereas the IRE1α/XBP1 axis is critical for the later step of antibody secretion.

### Loss of ARF4 arrests PB differentiation and causes a block in antibody secretion

Our screening data revealed that KO of *Arf4*, a small GTPase regulating COPI vesicle formation (47), predominantly affected PB formation. Recent evidence suggests that ARF4 protein exclusively localizes to the ER-Golgi intermediate compartment (ERGIC) in epithelial (HeLa) and myeloid (HAP1) cell lines, respectively (48), and seems to play a role in recycling of ER resident proteins back to the ER, thereby maintaining secretory capacity (49). Single KO of *Arf4* in HeLa cells causes secretion of ER resident proteins, indicating specific defects in coatomer-dependent retrieval by KDEL receptors of ER-resident proteins, such as the chaperones BiP, Calreticulin and GPR94 (encoded by *Hsp90b1*). Of note, KO of *Hsp90b1* affected PB differentiation in our screen (**Figure 1D**).

**Figure 4:**
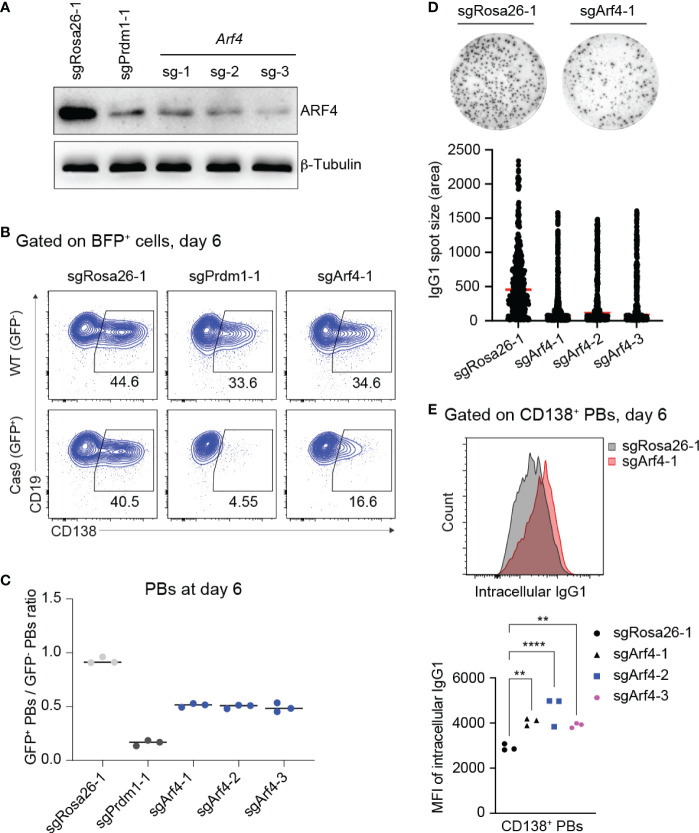
Loss of ARF4 arrests PB formation and blocks antibody secretion. **(A)** Western blotting for ARF4 protein showing gene KO efficiencies of sgRNAs targeting the *Arf4* gene. SgRosa26-1 and sgPrdm1-1 were used as negative and positive targeting controls. Beta-Tubulin was used as a loading control. **(B)** FACS analysis of PB differentiation six days post transduction, gates are set on CD138^+^ PBs in WT (GFP^-^, top) and Cas9 (GFP^+^, bottom) cells 6 days post transduction. **(C)** Scatter plot showing the ratio of GFP^+^ (Cas9)/GFP^-^ (WT) PBs corresponding to the experiment shown in **(B)** plus two biological repeat experiments. Each dot corresponds to one independent experiment averaged over two technical replicates; horizontal line indicates median. **(D)** Representative ELISPOT images showing antibody secretion in cells treated with indicated sgRNAs. Graph showing size (area) of IgG1 spots, quantified from a pool of 3 independent experiments. **(E)** Upper panel: Pre-gating on CD138^+^ PBs, FACS analysis showing expression levels of intracellular IgG1 in the cells targeted with sgRosa26-1 (grey) or sgArf4-1 (red). Lower panel: Scatter plot showing mean fluorescent intensity (MFI) of intracellular IgG1 levels in cells transduced with the indicated sgRNAs (statistically significant difference is indicated: two-way ANOVA, p values are indicated (**, P<0.01; ****, P<0.0001). n=3 independent experiments with two technical replicates each).

Therefore, we wondered whether loss *of Arf4* expression reduces antibody secretion by PBs in our cell culture system. First, we assessed gene KO efficiencies of sgRNAs targeting the *Arf4* gene. Based on western blotting, all sgRNAs targeting the *Arf4* gene led to efficient gene KO in Cas9-expressing B cells 6 days post retroviral transduction (**Figure 4A**). Consistent with our screening data, *Arf4* KO affected proliferation/survival only to a minor extent (similar to *Prdm1* KO; **Supplementary Figure 6**), while frequencies of CD138^+^ PBs were strongly reduced, although not to the same level as *Prdm1* KO (**Figures 4B, C**). Importantly, loss of ARF4 led to strongly impaired antibody secretion in CD138^+^ PBs (**Figure 4D**). To rule out that inefficient antibody secretion by *Arf4* KO PBs was due to decreased antibody synthesis, we measured the intracellular levels of IgG1 antibodies in *Arf4* KO and control (targeting with sgRosa26-1) PBs. *Arf4* KO PBs displayed a slightly higher level of intracellular IgG1 compared to control PBs (**Figure 4E**). Thus, our results suggest that the small GTPase ARF4, a member of the Ras-superfamily, plays a specific, non-redundant role in PB formation and facilitates efficient antibody secretion, possibly through ensuring chaperone recycling to the ER.

### Loss of HDAC1 impairs PB differentiation by perturbing the gene regulatory network that specifies PB versus B cell fate

**Figure 5:**
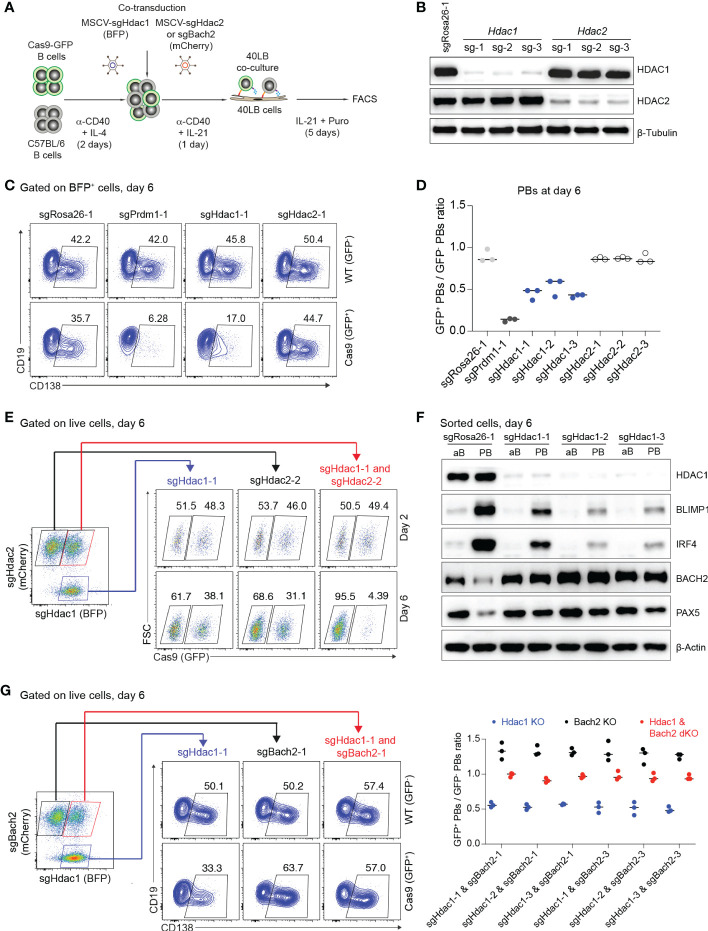
Loss of HDAC1 prevents PB differentiation by perturbing the gene regulatory network that specifies PB versus B cell fate. **(A)** Experimental scheme of co-transduction for activated B cells transduced with combinations of retroviral particles expressing *Hdac1*-targeting sgRNA (BFP^+^) and *Hdac2* or *Bach2*-targeting sgRNA (mCherry^+^). **(B)** Western blots for HDAC1 and HDAC2 proteins showing gene KO efficiencies of sgRNAs targeting *Hdac1* and *Hdac2* genes. SgRosa26-1 was used as a negative control. Beta-Tubulin used as a loading control. **(C)** FACS analysis of one technical repeat experiment at day 6 post transduction showing frequencies of CD138^+^ PBs in WT (GFP^-^, top) and Cas9 (GFP^+^, bottom) cells. **(D)** Scatter plot showing the ratio of GFP^+^ (Cas9)/GFP^-^ (WT) PBs corresponding to the experiment shown in **(C)** plus two independent repeat experiments. Each dot corresponds to one independent biological repeat experiment averaged over two technical replicates. **(E)** FACS analysis of one representative technical replicate of three independent experiments showing frequencies of GFP^+^ B cells in *Hdac1* KO (sgHdac1-1, blue), *Hdac2* KO (sgHdac2-2, black), or *Hdac1* and *Hdac2* double KO (dKO) (sgHdac1-1 and sgHdac2-2, red) cell populations in a single culture well, at day 2 and 6 post co-transduction. **(F)** Western blots showing expression levels of HDAC1, BLIMP1, IRF4, BACH2 and PAX5 transcription factors in sorted CD138^-^ activated B cells (aB) and CD138^+^ PBs (PB) treated with indicated sgRNAs. Beta-Actin was used as a loading control. **(G)** Pre-gating (left) on *Hdac1* KO (sgHdac1-1, blue), *Bach2* KO (sgBach2-1, black), or *Hdac1* and *Bach2* dKO (sgHdac1-1 and sgBach2-1, red) cell populations present in a single culture well for the FACS plot (middle) showing frequencies of CD138^+^ PBs in WT (GFP^-^, top) and Cas9 (GFP^+^, bottom) cells in each pre-gated cell population. Scatter plot (right) showing the ratio of GFP^+^/GFP^-^ PBs corresponding to the experiment shown on the middle (FACS analysis; one technical replicate of three) plus independent biological repeat experiments with different sgRNA combinations (n=3 independent experiments averaged over three technical replicates each; horizontal line indicates median).

The histone deacetylases HDAC1 and HDAC2 are catalytic core subunits of various deacetylase complexes and depending on the biological context may have partially overlapping or possibly distinct roles (50–53). In our screen, KO of *Hdac1, Mta2* and *Mbd2* (members of Mi-2/NurD complexes), but not KO of *Hdac2* affected PB differentiation, prompting us to more deeply analyze the roles of HDAC1 and HDAC2 in our *in vitro* PB differentiation system. All sgRNAs targeting *Hdac1* and *Hdac2* genes led to efficient gene KO (~80%) in Cas9-expressing B cells 6 days post transduction (**Figures 5A, B**). By measuring the expression of CD138, we confirmed that PB formation was consistently reduced by about 50% in *Hdac1* KO cells, while deletion of *Hdac2* had no effect (**Figures 5C, D**). In order to find out whether HDAC2 might partially compensate for HDAC1 in PB formation, we performed co-transduction experiments in which activated B cells (Cas9: WT mixture) were co-transduced with MSCV retroviral particles expressing sgRNAs targeting *Hdac1* (BFP^+^) or *Hdac2* (mCherry^+^) genes (**Figure 5A**). 6 days post co-transduction we observed a strong reduction of GFP^+^ B cells in *Hdac1*/*2* dKO cells, whereas loss of neither HDAC1 nor HDAC2 alone affected B cell survival or proliferation (**Figure 5E** and **Supplementary Figure 7**). This result is consistent with previous evidence that both HDAC1 and HDAC2 are required for B cell survival or proliferation (50). The strong effect on B cell survival or proliferation after deleting *Hdac1* and *Hdac2* genes together prevented further analysis for PB differentiation in this situation.

The reduction of PB formation in *Hdac1* KO cells suggests that the gene regulatory network that coordinates the GC B cell to PB transition may become disturbed. Two key transcriptional regulators that maintain B cell fate in GC B cells are BACH2 and PAX5 and they are antagonistically expressed relative to IRF4 and *Prdm1*/BLIMP1, two key transcriptional regulators that enforce the plasma cell gene expression program (9, 54). In order to investigate this issue, we sorted CD138^-^ activated Cas9-expressing B cells and CD138^+^ Cas9-expressing PBs 6 days post gene editing with sgRNAs targeting the *Hdac1* or control *Rosa26* gene. Based on protein blotting assays, deletion of HDAC1 led to a decreased expression of IRF4 and BLIMP1, and a strongly increased expression of BACH2 and PAX5 in CD138^+^ PBs (**Figure 5F**), suggesting that full repression of the B cell program cannot be achieved in *Hdac1* KO cells differentiating towards PBs.

HDAC1 directly represses the expression of BACH2 in activated T cells (55), thus, a similar mechanism might also operate in activated B cells, particularly when differentiating to PBs. Therefore, we wondered whether deletion of BACH2 might restore PB differentiation in *Hdac1* KO cells. To address this point, we performed co-transduction experiments to generate *Hdac1* single KO cells (BFP^+^), *Bach2* single KO cells (mCherry^+^) or *Hdac1*/*Bach2* double KO (dKO) cells (BFP^+^ mCherry^+^) in the same culture well (**Figures 5A, G**). First, consistent with previous evidence (56, 57), we observed a significant increase in CD138^+^ PBs in *Bach2* single KO cells, presumably because BLIMP1 becomes upregulated in the absence of its repressor BACH2 and facilitates PB formation (56). Second, PB formation was completely rescued in *Hdac1*/*Bach2* dKO cells comparing to *Hdac1* single KO cells (**Figure 5G**), indicating that like in activated T cells, HDAC1 might directly repress the expression of BACH2 during PB formation. Thus, we uncovered a specific, non-redundant role of the histone deacetylase HDAC1 in PB differentiation, possibly through direct repression of BACH2.

## Discussion

In order to gain new insights into the molecular determinants underlying the transition from pre-PBs to PBs, an early step in plasma cell differentiation that is characterized by the surface expression of the traditional plasma cell marker CD138/syndecan-1 (8, 11, 58), we took advantage of our previously established CRISPR/Cas9-based screening system (18) that models T cell-dependent plasma cell differentiation *in vitro* (20). Out of the 568 candidate genes selected for high expression in plasma cells, as many as 133 genes strongly decreased B cell survival or proliferation, indicating that these genes are essential when activated B cells develop into professional antibody secreting cells. However, our screen also identified known and previously unknown genes and signaling pathways that predominantly influenced PB formation.

Terminal differentiation of B cells toward antibody-secreting plasma cells requires the expansion of the secretory apparatus and other organelles to adapt the cells to an increased antibody load (44). This functional and morphological change occurs after the acquisition of surface CD138 expression (45) and is controlled by the transcription factor XBP1s, a downstream UPR effector of the ER stress sensor IRE1α which converts an unstable XBP1 protein to a stable, highly active form through an unusual mRNA splicing mechanism (59). In this study, we discovered that the formation of CD138^+^ PBs requires the activities of both, IRE1α’s catalytic kinase and endonuclease domains, but is unaffected by XBP1 KO. Thus, we uncovered an additional, previously unknown function of IRE1α that plays a role in the developmental window between acquisition of CD138 expression and the full development of the plasma cell secretory apparatus.

Disturbance of ER homeostasis or the secretory pathway at this early stage appears to lead to an inhibition of plasma cell differentiation. In line with this, ARF4, a small GTPase that localizes to the ERGIC and regulates secretory vesicle formation is upregulated in activated B cells in an IRE1α -dependent manner (**Supplementary Figure 4**). In epithelial cells, ARF4 plays a role in retrieval and thus continuous replenishment of ER resident chaperones such as BiP, Calreticulin and GPR94/*Hsp90b1* (48). Similar to KO of IRE1α, KO of ARF4 and GRP94/*Hsp90b1* arrested PB formation. Loss of ARF4 blocked antibody secretion, but not antibody formation per se. As a consequence, the increase of accumulated antibodies in secretory organelles such as the ER might trigger UPR-associated apoptosis in these cells (60).

Our screen also revealed that KO of several components of Mi-2/NuRD complexes affected PB formation. Out of 14 components surveyed (*Hdac1/2, Mta1/2/3, Mbd1/2/3, Chd3/4, Gatad2A/B, Rbbp4/7)*, three showed significant (*Hdac1*, *Mta2* and *Mbd2*), and one borderline effects (*Rbbp4)*. We more deeply investigated the consequences of the deletion of the *Hdac* genes, and found that double KO of *Hdac1/2* severely impaired survival or proliferation of activated B cells, while single KO of *Hdac1* selectively affected plasma cell differentiation, and single KO of *Hdac2* had no effect (**Figure 5** and **Supplementary Figure 7**). These findings add to a growing body of evidence, that “distinct permutations of the NuRD complex with tailored functions might regulate tissue-specific gene expression programs” (51).

NuRD complexes control multiple stages of B cell development and GC B cell identity through interactions with the transcription factors IKAROS, AIOLOS and BCL6, respectively (61). In human GC B cells, BCL6, the transcriptional master regulator of GC B cell identity interacts with the NurD components MBD3, HDAC1, MTA3, and CHD4 to block terminal differentiation toward the plasma cell fate (62). IKAROS and AIOLOS control the development of long-lived plasma cells (63, 64). Interestingly, both IKAROS and AIOLOS interact with the MTA2 component of NurD complexes (65), raising the possibility that NurD complexes may be involved in the regulation of plasma cell generation. Moreover, BLIMP1 interacts with various components of NurD complexes such as HDAC1, HDAC2 and LSD1, and acts as a transcriptional repressor that initiates plasma cell differentiation, through repression of the B cell transcription factors PAX5, BACH2, CIITA, ID3 and SPIB (11, 66–68). Our results suggest that in activated B cells, similar to the situation in activated T cells (55), HDAC1 may exert its role in promoting plasma cell differentiation through repression of BACH2, a repressor of BLIMP1. However, the exact mechanism of how HDAC1 modulates plasma cell differentiation, and whether it does so as part of a developmental stage-specific NuRD complex needs to be further elucidated.

Limitations of this study: *In vivo*, plasma cells emerge through different developmental pathways (extrafollicular or in GCs), depending on the B cell type from which they originate, the activating antigen, and the affinity of the B cell receptor, and consequently acquire different properties relating to location, survival, and the quality and quantity of the secreted antibodies (2, 8). By its very nature, an *in vitro* culture system can address only certain aspects of the much more complex *in vivo* situation. In case of CRISPR/Cas9-mediated KO of *Arf4* and *Hdac1*, as well as for several transcription factors known to be involved in plasma cell formation (*Irf4*, *Pou2af1* (OBF-1), *Sp3*, *Tcf3* (E2A) and *Stat3*), we consistently observed only a 50% or less reduction in PB formation. The reasons for this incomplete penetrance of the KO genotypes are unclear and remain to be determined. They could be due to the heterogenous B cell populations present in our culture system such as distinct spleen-derived B cell subsets, distinct differentiation pathways or the non-synchronized differentiation conditions. We also note that in our 40LB *in vitro* culture system, after Blimp1/*prdm1*-KO, B cells did not show increased survival or proliferation, while it has been reported that *prdm1* ^-/-^ B cells stimulated with LPS are hyperproliferative, possibly owing to the different culture conditions (69).

Within these limitations, our *in vitro* screening approach has proved to be a useful high-throughput platform suitable for identifying new molecular players in the T-cell dependent transition from B cells to early plasmablasts. *In vivo* validation of such molecules might lead to the discovery of new drug targets in diseases involving deregulated plasma cell differentiation such as Lupus and plasmacytoma.

## Data availability statement

The mass spectrometry proteomics data presented in this study are deposited to the ProteomeXchange Consortium via the PRIDE partner repository [1] with the dataset identifier PXD038201. The data can be found here: http://www.ebi.ac.uk/pride/archive/projects/PXD038201.

## Ethics statement

The animal study was reviewed and approved by Institution Animal Care and Use Committee.

## Author contributions

VC and KR designed the study. VC, EX and CS performed experiments and acquired data. VC, EX, OP, PM, CK and KR analyzed and interpreted data. OP and PM carried out the proteomics analysis. VC, EX, CK and KR wrote the paper. All authors contributed to the article and approved the submitted version.

## Supplementary material

Supplementary Table 1 List of selected candidate genes highly expressed in plasma cells.

Supplementary Table 2 List of used sgRNAs.

Supplementary Table 3 Correlation of survival/proliferation and differentiation scores.

Supplementary Table 4 Proteins are significantly up- and down-regulated in IRE1a KO cells.
