# Supplementary material for: A CRISPR/Cas9-mediated screen identifies determinants of early plasma cell differentiation
Source: Front Immunol. 2023 Jan 5;13:1083119. doi: 10.3389/fimmu.2022.1083119 (PMC9849354; doi:10.3389/fimmu.2022.1083119)
Supplement: Supplementary file 5 [file DataSheet_1.docx]

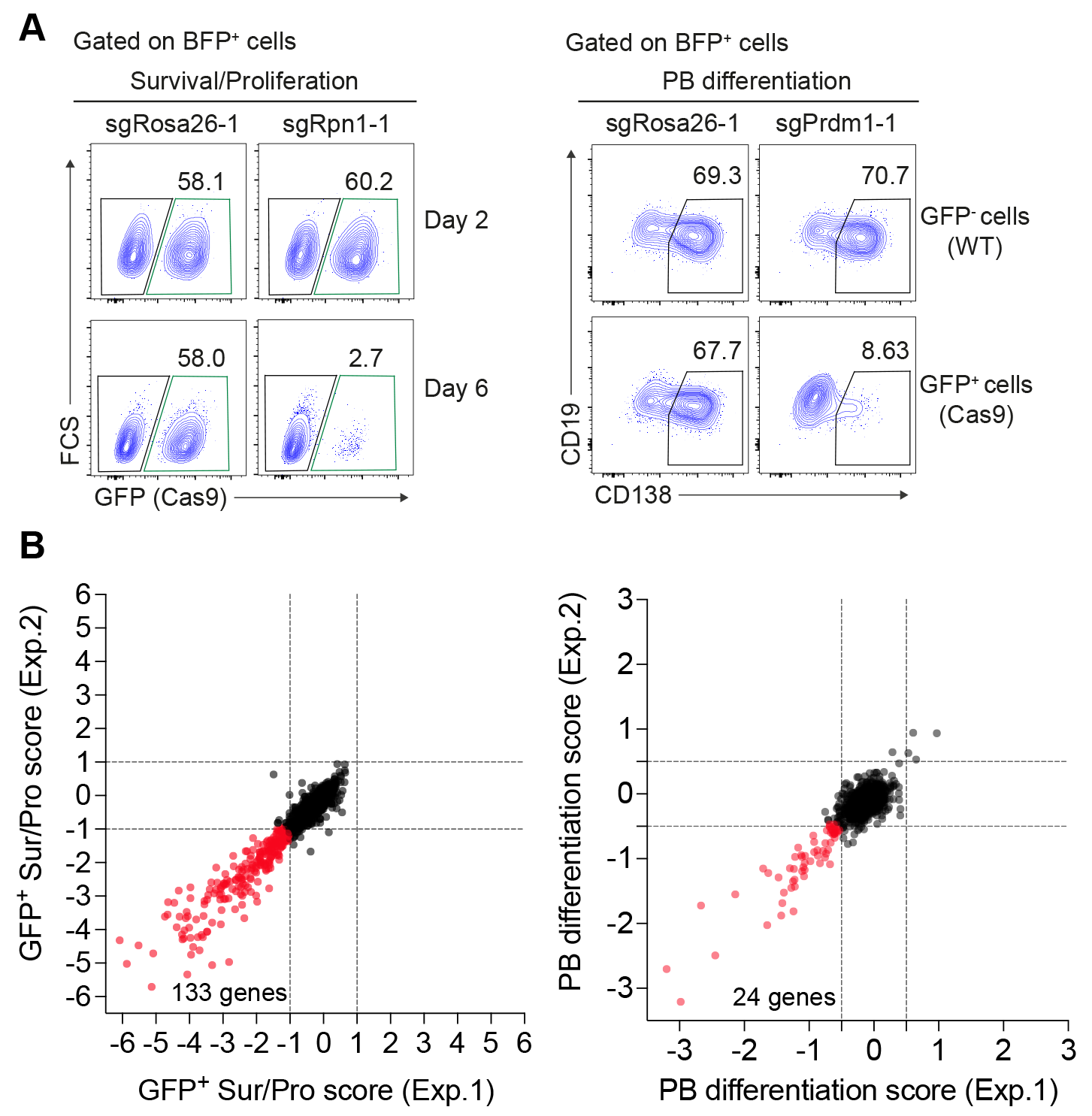


**Supplementary Figure 1. CRISPR/Cas9-based screen for B cell survival/proliferation and PB differentiation.**

(**A**) Left: Pre-gating on BFP^+^ cells, FACS analysis showing the percentage of GFP^+^ B cells targeted with sgRosa26-1 (control) or sgRpn1-1 (targeting Ribophorin) at day 2 and day 6 after transduction. Right: FACS analysis of PB differentiation, indicated by the expression of CD138 in GFP^-^ (WT, top) and GFP^+^ (Cas9, bottom) cells targeted with sgRosa26-1 or sgPrdm1-1 (targeting BLIMP1) at day 6 after transduction. (**B**) Correlation graphs showing survival/proliferation (left) and differentiation (right) scores for two independent experiments. The cutoffs of log2 fold change for differentiation (<-0.5) and survival/proliferation (<-1) scores are indicated. 133 genes affecting B cell survival/proliferation and 24 genes affecting PB differentiation above the cutoffs are highlighted in red.


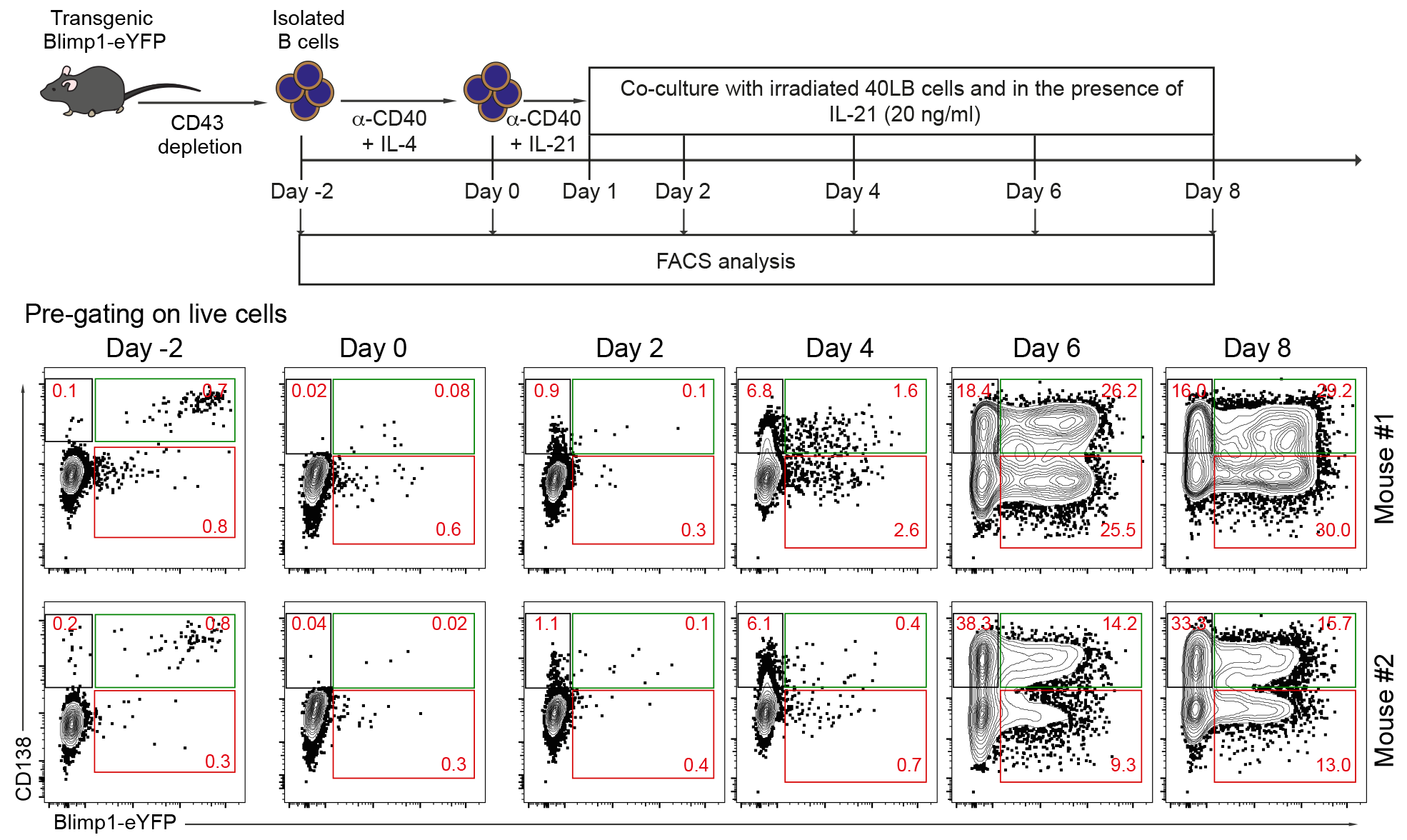


**Supplementary Figure 2. Dynamics of PB differentiation in the 40LB system as monitored by expression of a Blimp1-YFP reporter.**

Upper part: Experimental scheme. B cells were isolated from the spleens of two individual Blimp1-YFP mice by depletion of CD43-positive cells. To mimic retroviral transduction experiments shown in Fig. 1A, the cells were activated with anti-CD40 antibody and IL-4 for 2 days, followed by anti-CD40 antibody and IL-21 for one day and subsequent co-culture on irradiated 40LB feeder cells in the presence of IL-21. Percentages of PBs were monitored at different time points using flow cytometry. Lower part: Pre-gating on live cells, FACS plots showing frequencies of CD138^+^ (black), Blimp1-YFP^+^ (red), and CD138^+^ Blimp1-YFP^+^ (green) PB populations from individual mice. At Day 0, corresponding to the time point of retroviral transduction and up to 2 days after transduction, only background levels of CD138^+^/Blimp1-YFP^+^ double-positive cells were detectable, confirming that the vast majority of activated B cells was not differentiating into PB at the time of transduction (Day 0) or the timepoint of transfer to 40LB feeder cells (Day 1).

**Supplementary Figure 3. Analysis of B cell survival/proliferation for ER sensors and UPR pathway effectors.**


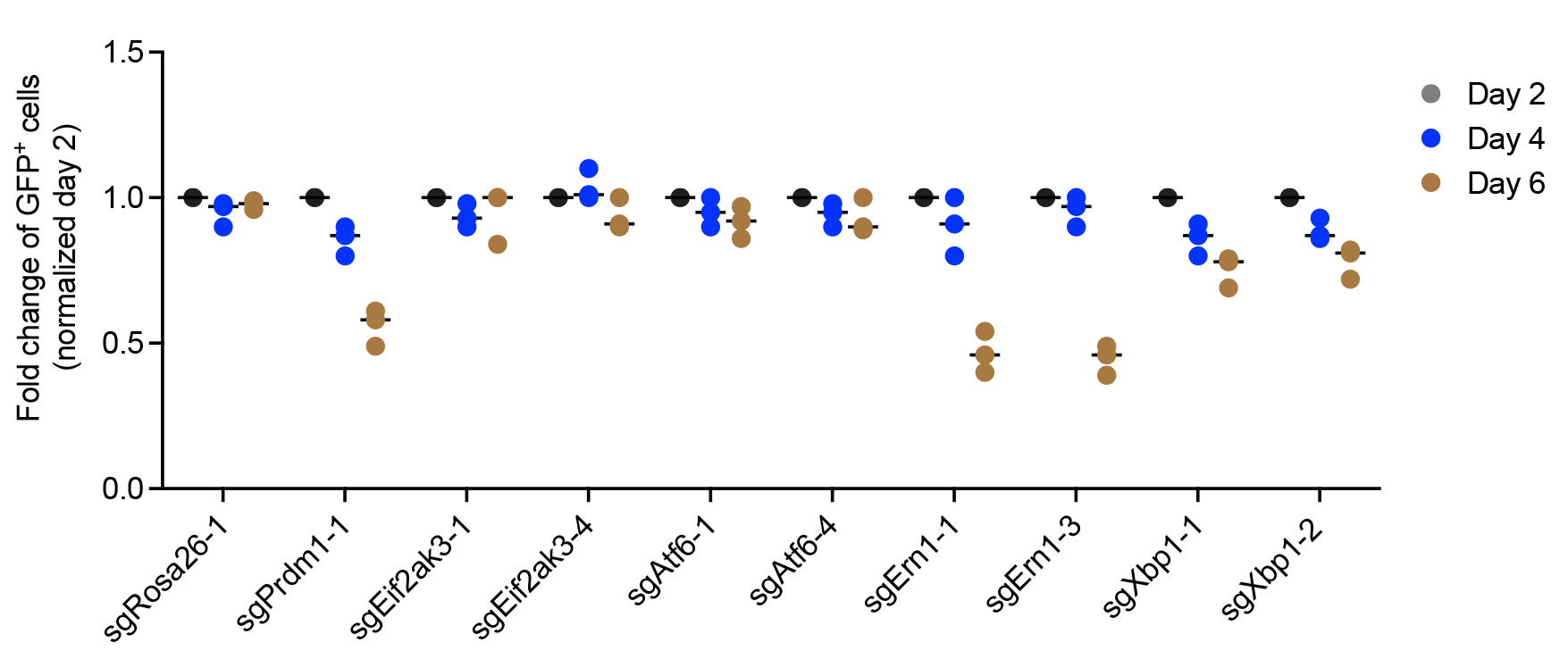


Graph showing the fold change of GFP^+^ B cells targeted with the indicated sgRNAs (directed against ER sensor or UPR pathway components) and analyzed the indicated time points, normalized to day 2 post transduction. Data are shown as mean ± SD (n=3 independent experiments).


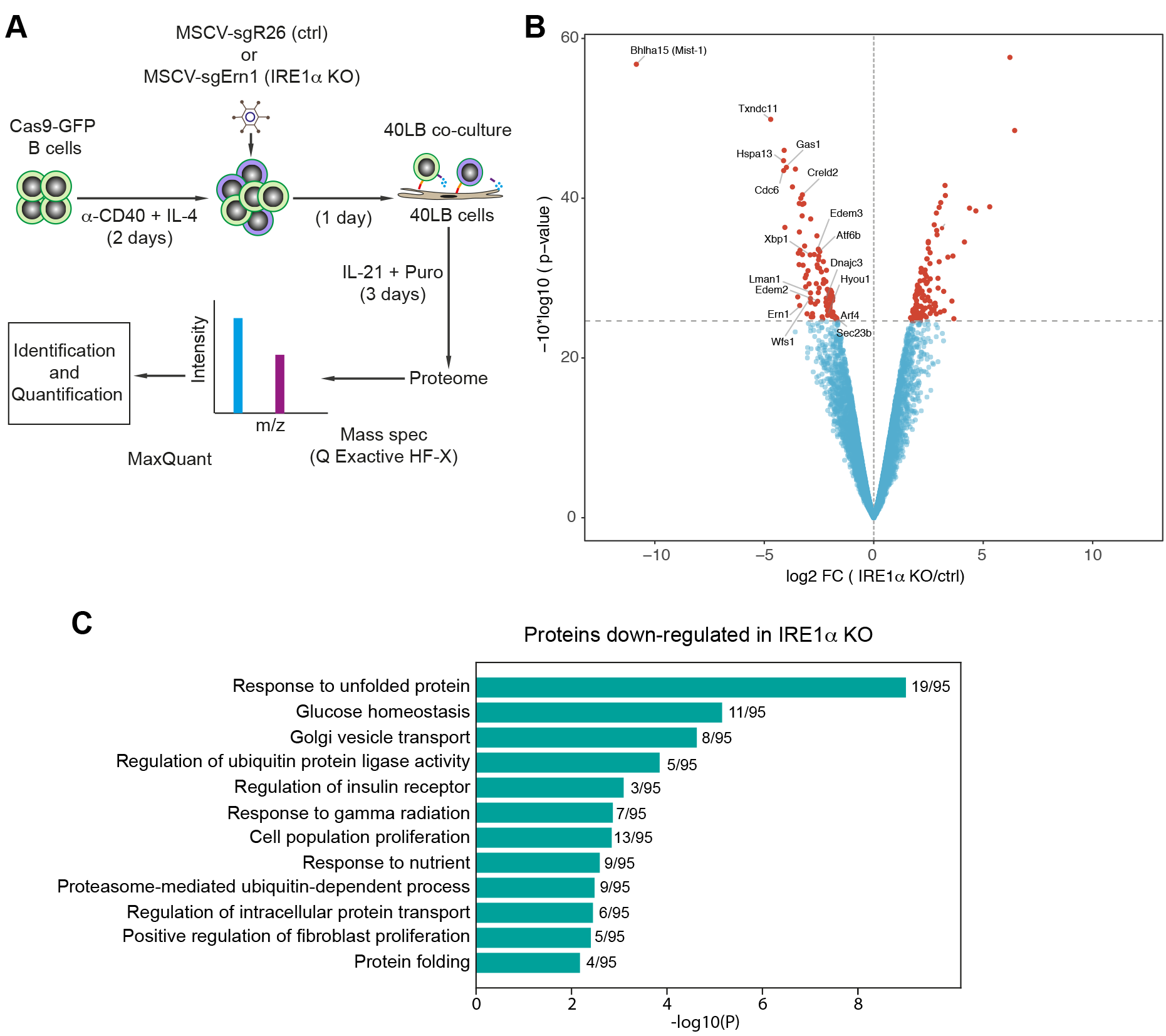


**Supplementary Figure 4. Activated B cells express effectors of the XBP1s branch of the UPR.**

(**A**) Scheme of proteomics experiments for activated B cells 4 days post transduction (IRE1α KO (sgErn1) and control (sgRosa26-1)). In order to reduce the amount of dying cells in the cell preparation (generated by apoptosis due to “frustrated” PB differentiation), we modified our original protocol (Figure 1A) by omitting the addition of IL-21 cytokine in the 1-day culture period before transferring cells to irradiated 40LB feeder cells (expressing CD40L and BAFF). With this modification, the fraction of CD138^+^ PBs in the culture was less than 2% at day 4. (**B**) Volcano plot showing significantly up- (110) or down-regulated (95) proteins in IRE1α KO versus control (ctrl) B cells. Up- and down-regulated proteins are highlighted in red. Among 95 down-regulated proteins in IRE1α KO cells, IRE1α (Ern1), ARF4, XBP1 and its target proteins were labelled, respectively. (**C**) GO enrichment analysis showing biological processes of 95 down-regulated proteins in IRE1α KO cells. Number of proteins enriched in each biological process was indicated (analysis performed using Metascape software (https://metascape.org/)).


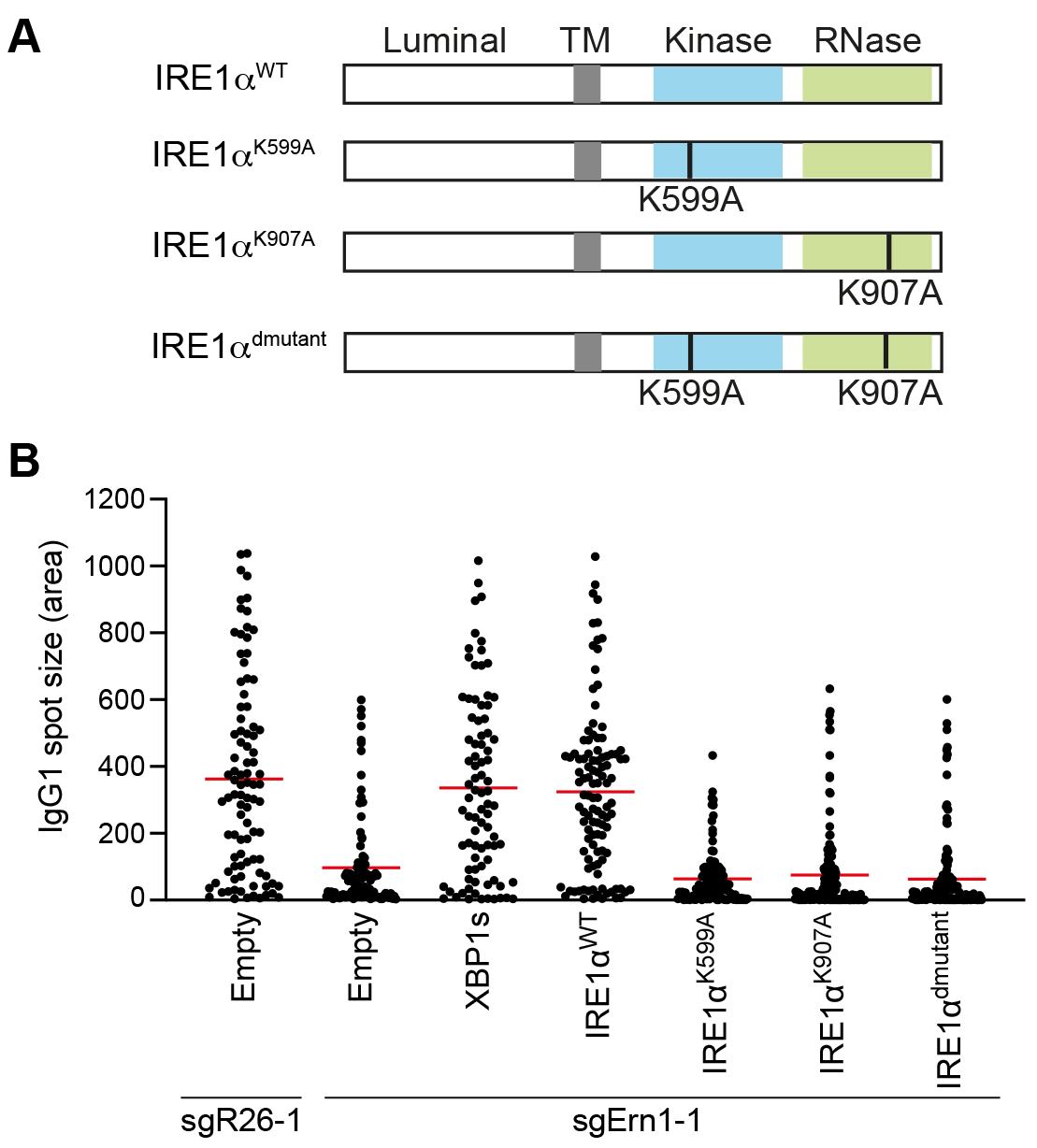


**Supplementary Figure 5. Antibody secretion of IRE1α KO cells could be rescued by over-expressing XBP1s and IRE1α^WT^ but not catalytically inactive forms of IRE1α.**

(**A**) Scheme depicting luminal, trans-membrane (TM, grey), kinase (light blue) and ribonuclease (RNase, green) domains of IRE1α protein. K599A and K907A mutations in kinase and RNase domains are highlighted, respectively. (**B**) Graph quantifying the size (area) of IgG1 spots in IRE1α KO (sgErn1-1) and control (sgRosa26-1 (sgR26-1)) sorted PBs over-expressed with indicated cDNAs (data pooled from three independent experiments).


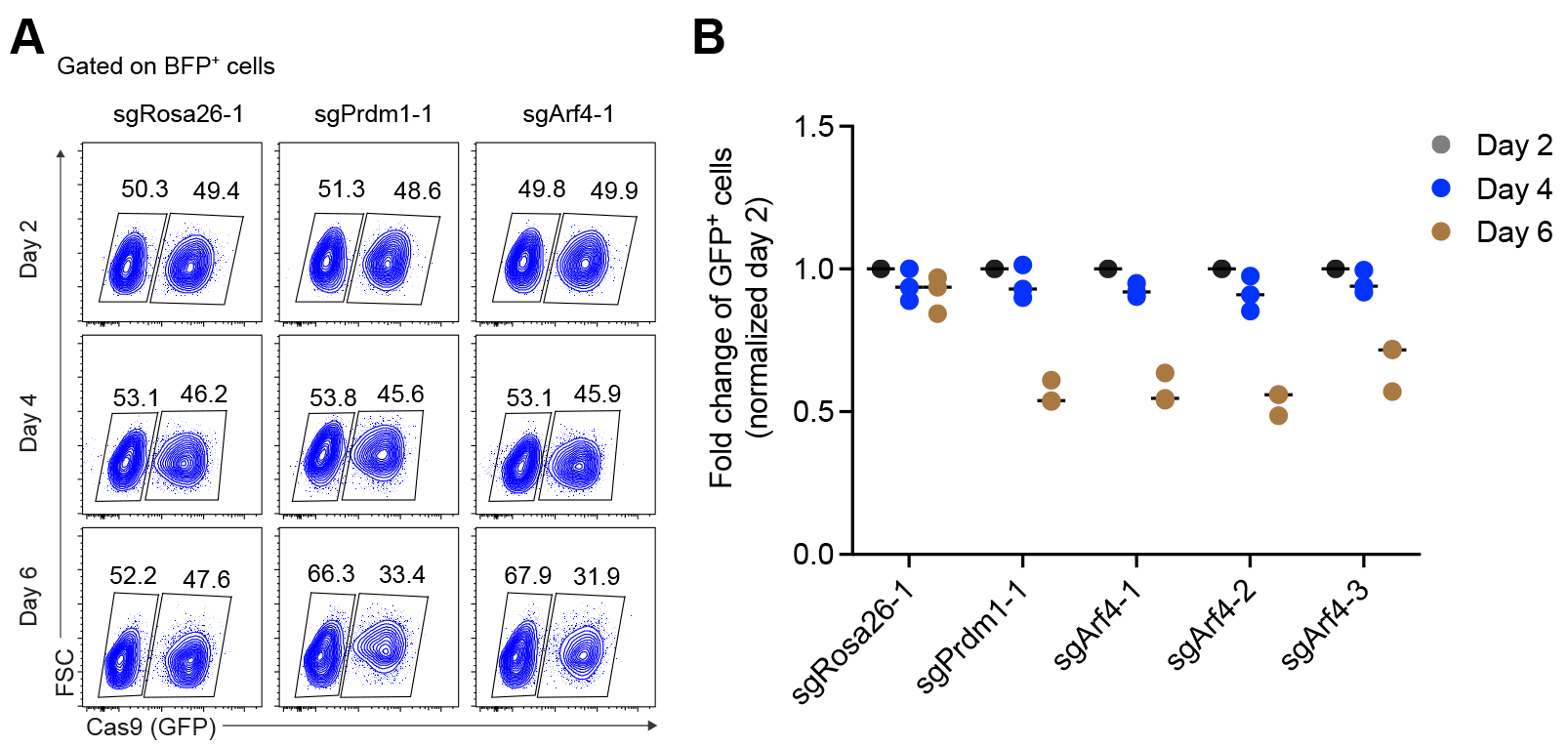


**Supplementary Figure 6. CRISPR-mediated *Arf4* KO has only minor effects on B cell survival/proliferation at a late timepoint (day 6) (comparable to *Prdm1*/BLIMP1 KO).**

**(A)** Pre-gating on BFP^+^ transduced B cells, FACS analysis showing frequencies of GFP^+^ B cells treated with indicated sgRNAs at different time points. (**B**) Graph summarizing fold change of GFP^+^ B cells as in (**A**). Data are shown as mean ± SD (n=3 independent experiments).


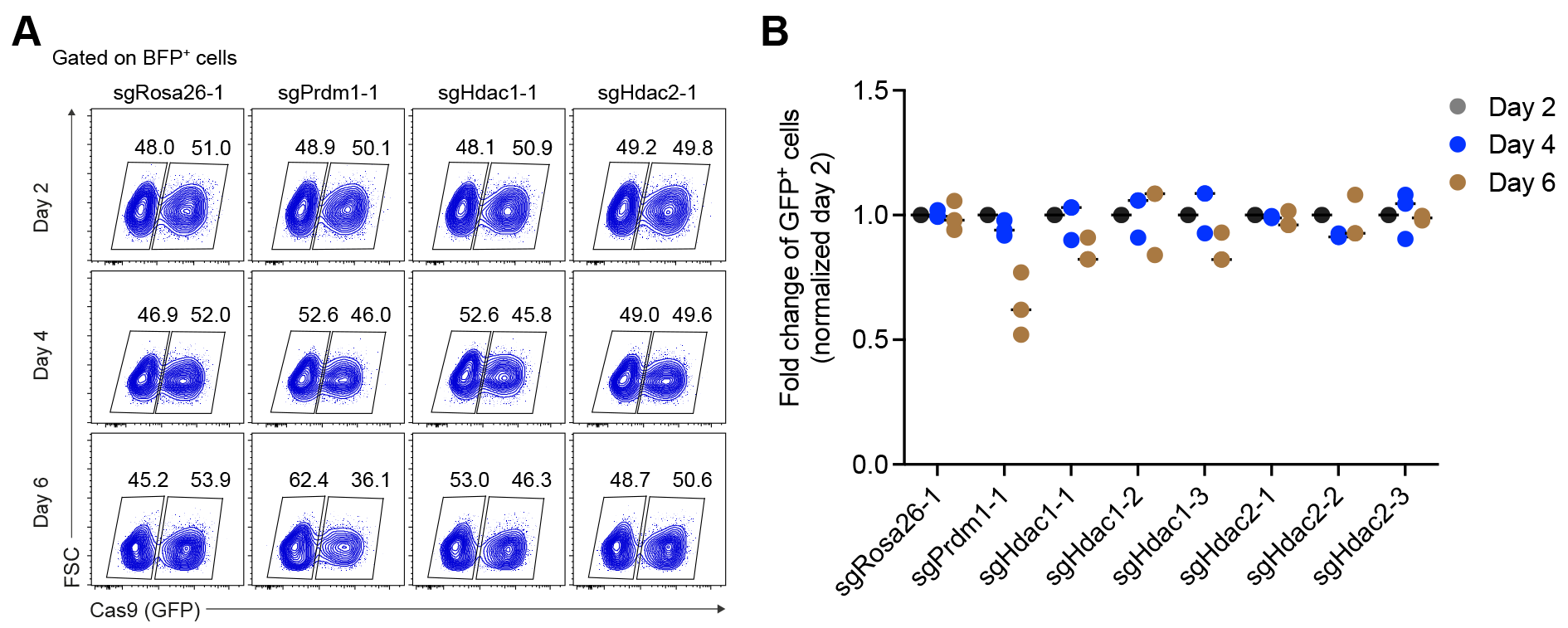


**Supplementary Figure 7. CRISPR-mediated KO of *Hdac1* and *Hdac2* does not affect B cell survival/proliferation.**

**(A)** Pre-gating on BFP^+^ transduced B cells, FACS analysis showing frequencies of GFP^+^ B cells treated with indicated sgRNAs at different time points. (**B**) Graph summarizing fold change of GFP^+^ B cells as in (**A**). Data are shown as mean ± SD (n=3 independent experiments).
